# Sulfonal in Insomnia

**Published:** 1892-10

**Authors:** 


### SULFONAL IN INSOMNIA.

The recent reports concerning the value of this hypnotic agent, affirm the earlier clinical conclusions that Sulfonal must be regarded as our best hypnotic. One or two observers cite some instances in which drowsiness has followed its use, and two cases are mentioned in which dizziness occurred after a sleep of ten hours produced by Sulfonal. The reported details seem to show that the doses administered were somewhat in excess of the amount of Sulfonal necessary to obtain a useful therapeutic effect. The weight of evidence, as shown in the very large number of cases thus far reported, would seem to demonstrate that Sulfonal is an active hypnotic, remarkably free from toxic influences of any kind, especially available for the exigencies of general practice in the treatment of all forms of insomnia.

The value of Sulfonal in the neuroses has been widely tested by neurologists, whose published conclusions point to a wide field for the use of Sulfonal in this direction. In asylum practice Sulfonal has largely replaced the opiates and narcotics, and the reports agree that its use was not followed by the pre-somnial excitation and post-somnial exhaustion which so often supervenes upon the administration of other sleep-producing remedies. It was noted also that the good effects of Sulfonal often continued for many days after it was withdrawn.

The question as to the liability of Sulfonal to induce a drug habit has been considered by several investigators, all of whom report to the effect that such a sequence would be unlikely to follow a remedy of this nature. One observer presents the reason for this, as follows: "Drugs which give rise to habits, are those which, like opium and cocaine, stimulate before they effect narcosis, and habitues take them for their qualities as incitant; Sulfonal is a pure hypnotic, and gives that effect alone." This seems to formulate the consensus of medical opinion upon this subject.—*Exchange.*

